# Supplementary material for: Drug repurposing for aging research using model organisms
Source: Aging Cell. 2017 Jun 16;16(5):1006–15. doi: 10.1111/acel.12626 (PMC5595691; doi:10.1111/acel.12626)
Supplement: Supplementary file 7 — Data S1 Zip‐Archive of all report cards. [file ACEL-16-1006-s007.zip › RC_X2L.pdf]

X2L

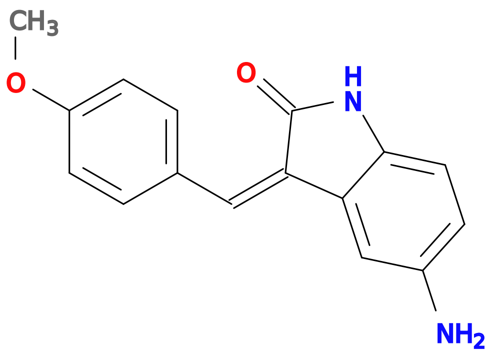

Database identifiers

ChEMBLCompound CHEMBL591696

Ranking

	Rank	Score
Drosophila	646/697	0.113
C. elegans	NA	NA

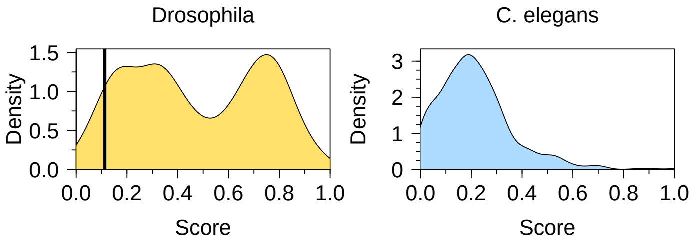

	Ageing implication	Domain conservation	Binding site conservation	Binding affinity	Bioavailability	Lipinski	Promiscuity	Purchasability	Drug approval	**Total**
Drosophila	0.203	0.909	0.957	0.714	(0.9)	0.0	-0.0	0.0	0.0	0.113
C. elegans	NA	NA	NA	NA	NA	NA	NA	NA	NA	NA

Names

No synonyms found

Roles

ChEBI entry None has no roles

Status

Approved drug (according to ChEMBL)	No
Number of Rule of 5 violations	0
Binding affinity to original target in log units (RF-Score prediction)	5.92
Burns *C. elegans* bioavailability prediction	0.59

Compound Target Characteristics

Proto-oncogene tyrosine-protein kinase receptor Ret

Best gene implication in ageing for this target family came from gene P07949 via mapping the annotation from Ensembl ENSG00000165731 via mapping the annotation from EntrezGene 5979 via mapping the annotation from GenAgeHuman 0056 annotated in GenAge release 17.

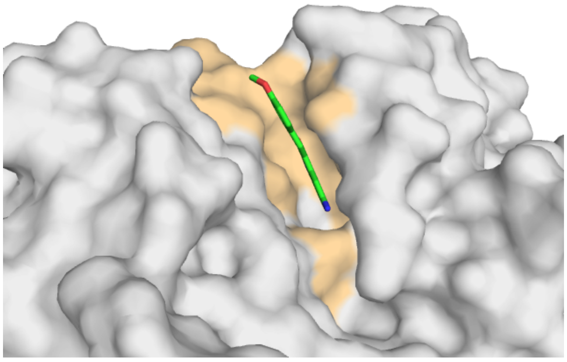

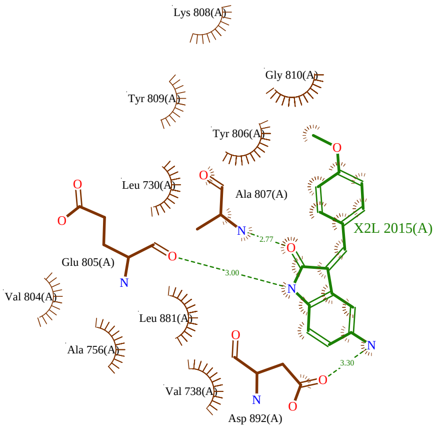

protein	amino acids contacts (binding site)
PDB:2x2l:chainA:P07949	L V A V E Y A K Y G L D
tr:B4DGX8:B4DGX8_HUMAN	L V A V E Y A K Y G L D
sp:P07949:RET_HUMAN	L V A V E Y A K Y G L D
sp:G3V9H8:RET_RAT	L V A V E Y A K Y G L D
sp:P35546:RET_MOUSE	L V A V E Y A K Y G L D
tr:Q7JQD5:Q7JQD5_DROME	L V A I E Y A R Y G L D
tr:Q7KA88:Q7KA88_DROME	L V A I E Y A R Y G L D
tr:Q7KT06:Q7KT06_DROME	L V A I E Y A R Y G L D
tr:Q9BHA9:Q9BHA9_DROME	L V A I E Y A R Y G L D

	whole protein		domain-based		contact-based	
protein	ident	simil	ident	simil	ident	simil
PDB:2x2l:chainA:P07949	0.99	1.0	1.0	1.0	1.0	1.0
tr:B4DGX8:B4DGX8_HUMAN	0.73	0.73	1.0	1.0	1.0	1.0
sp:P07949:RET_HUMAN	1.0	1.0	1.0	1.0	1.0	1.0
sp:G3V9H8:RET_RAT	0.85	0.95	0.96	0.99	1.0	1.0
sp:P35546:RET_MOUSE	0.83	0.95	0.95	0.99	1.0	1.0
tr:Q7JQD5:Q7JQD5_DROME	0.17	0.39	0.55	0.83	0.83	0.96
tr:Q7KA88:Q7KA88_DROME	0.2	0.6	0.55	0.83	0.83	0.96
tr:Q7KT06:Q7KT06_DROME	0.2	0.6	0.55	0.83	0.83	0.96
tr:Q9BHA9:Q9BHA9_DROME	0.2	0.6	0.55	0.83	0.83	0.96

Ret (FBgn0011829) associated phenotypes

decreased cell death, heat sensitive, lethal - all die before end of pupal stage, partially lethal - majority die, size defective, somatic clone

(Information from FlyBase)
